# Astragaloside IV protects human cardiomyocytes from hypoxia/reoxygenation injury by regulating miR-101a

**DOI:** 10.1007/s11010-020-03743-5

**Published:** 2020-05-11

**Authors:** Yang Wu, Zongjing Fan, Zhengju Chen, Jiqiang Hu, Jie Cui, Yang Liu, Yao Wang, Bin Guo, Juan Shen, Liandi Xie

**Affiliations:** 1 Department of CardiologyDong Fang HospitalFengtai District, Beijing University of Chinese Medicine, No. 1 Chang Xin Dian Chen Zhuang Avenue, Beijing, 100078 China; 2 Technical Consultant Department of Technology Center, Beijing 100Biotech Co., Ltd, Beijing, 100078 China; 3 Beijing University of Chinese Medicine, Beijing, 100078 China

**Keywords:** Hypoxia/reoxygenation injury, Astragaloside IV, MiR-101a, MAPK signaling pathway

## Abstract

Astragaloside IV (AS/IV) is one of the extracted components from the traditional Chinese medicine Astragalus which has been demonstrated to have potential capacity for anti-inflammation activity and for treating cardiovascular disease. Our purpose was to determine the function and underlying molecular mechanism of AS/IV in hypoxia/reoxygenation (H/R) injured in cardiomyocytes. Differentially expressed genes (DEGs) were screened using bioinformatic analysis, and the molecular targeting relationship was verified by the dual-luciferase report system. H/R injured cardiomyocytes were employed to explore the effect of AS/IV. QRT-PCR and Western blot analysis were applied to detect the expression of mRNA and proteins, respectively. Additionally, superoxide dismutase (SOD), lactic dehydrogenase (LDH) and MDA (malondialdehyde) levels were detected to determine the oxidative damage. Cell viability was assessed by CCK-8, and flow cytometry was used to evaluate cell apoptosis ratio. *TGFBR1* and *TLR2* were selected as DEGs. Additionally, AS/IV could enhance cell proliferation and upregulated miR-101a expression, which suppressed *TGFBR1* and *TLR2* expression in H/R injured cardiomyocytes. Moreover, the results of Western blot exhibited that the downstream genes (p-ERK and p-p38) in the MAPK signaling pathway were suppressed, which meant AS/IV could inhibit this pathway in H/R injured cardiomyocytes. Overall, this study demonstrated AS/IV could attenuate H/R injury in human cardiomyocytes via the miR-101a/*TGFBR1*/*TLR2*/MAPK signaling pathway axis, which means that it could serve as a possible alternate for H/R treatment.

**Electronic supplementary material:**

The online version of this article (10.1007/s11010-020-03743-5) contains supplementary material, which is available to authorized users.

## Introduction

Myocardial ischemia–reperfusion injury (I/RI) is the main reason for mortality among heart disease patients [1]. Ischemia and subsequent reperfusion result in damage to the myocardium during acute myocardial infarction. Myocardial hypoxia/reoxygenation (H/R) possesses sophisticated associations with many molecules that all contribute to the final damage inflicted on the heart [2]. In the past decade, many therapeutic agents have been reported to alleviate myocardial H/R injury, including clematichinenoside, ammonium tetrathiomolybdate and abciximab [2–4]. However, the results of clinical trials were unsatisfactory. Therefore, new cardioprotective agents are urgently needed to improve therapeutic outcomes and reduce myocardial H/R injury.

Astragaloside IV is the main active ingredient extracted from the Astragalus membranaceus, a Chinese herb [5]. Recent studies have revealed that AS/IV has a cardioprotective capacity and interacts with several molecules. For instance, Lu et al*.* showed that AS/IV alleviated myocardial H/R injury in rats by modulating the toll-like receptor 4/nuclear factor-kappaB signaling pathway [6]. Huang et al*.* provided evidence for the protective ability of AS/IV for cardiomyocytes stemming from the anoxia/reoxygenation injury through upregulation of Hes1 protein expression [5]. Additionally, we wanted to further clarify the molecular mechanisms through which AS/IV can play a role in myocardial H/R.

MicroRNAs (miRNAs) are a kind of endogenous, conserved, noncoding small RNAs with lengths of 20–25 nucleotides [7]. It has been demonstrated that numerous miRNAs were associated with myocardial IR/I [8]. For example, Inhibition of miR-192 expression can significantly protect myocardial I/R injury after myocardial infarction in rats [9], and miR-320 can regulate myocardial cell apoptosis induced by ischemia reperfusion injury by target binding to AKIP1 [10]. In our studies, we sought to explore the effects of miR-101a on H/R cells.

Transforming growth factor beta (TGF-beta, TGFβ) is a bifunctional regulator that either inhibits or stimulates cell proliferation, and it has three isoforms (*TGFβ2*, *β2*, and *β3*) [11]. *TGFβ* was vitally important in wound healing and fibrosis as well as the negative modulation of inflammation [12–14]. When the TGFβ signaling pathway is activated, TGFβ first binds to the TGFβ type 2 receptor (TGFBR2) on the outer membrane of a cell, then the type I receptor (TGFBR1) was activated. After that, the Smad protein is activated by phosphorylation. In the end, the activated Smad complexes are transported to the nucleus to regulate gene transcription [15]. Previous studies have reported that TGFBR1 plays a key role in myocardial injury. For example, Chen et al. proved that the A83-01 blocked the TGF signaling pathway by inhibiting TGFBR1 to protect heart function in mice with myocardial injury [16]. Cheng et al. also reported that miR-98 protects TGFβ1-induced myocardial fibrosis by targeting and inhibiting TGFBR1 [17]. In addition, it has been reported that the activation of TGFBR1 and TGFBR2 can activate the MAPK cascade and improve the phosphorylation levels of p38, JNK1/2 and ERK1/2 [18].

Toll-like receptors (Toll-like receptors, TLRs) are important protein molecules involved in nonspecific immunity, which is a transmembrane pattern recognition receptors [19]. The activation of TLRs can increase inflammation after ischemia reperfusion injury [20]. TLR2 is a member of the TLRs family which plays an important role in myocardial ischemia–reperfusion injury [21]. Arslan et al. confirmed that the inhibition of TLR2 by OPN-301 can significantly retrieve myocardial I/R injury and protect cardiac function [22]. Moreover, Yang et al*.* have reported that the decreased expression of miR-101 can aggravate the development of rheumatic heart disease by upregulating TLR2 [23]. However, the molecular mechanism of TLR2 in myocardial ischemia reperfusion remains to be investigated.

Mitogen-activated protein kinases (MAPKs) are serine–threonine kinases that regulates various intracellular signaling pathways associated with cell life activities, including intercellular signaling, cell apoptosis, cell proliferation, cell differentiation and other cell activities [24]. MAPKs are also involved in numerous diseases. Xiao proved that miR-125b attenuates the carcinogenic progression of osteosarcoma cells by regulating the MAPK-STAT3 signaling pathway [25], and Han et al*.* confirmed that microRNA-128 contributed to gastric carcinoma progression by activating GAREM-mediated MAPK signaling [26]. In the hypoxia/reoxygenation injury model, antiphospholipid antibody can affect the apoptosis of neonatal rat cardiomyocytes by regulating the p38 MAPK signaling pathway [27]. Based on prior investigations, we sought to explore the potential mechanism of AS/IV activity through the MAPK signaling pathway in myocardial H/R injury.

This study targeted the effect and efficacy of AS/IV on myocardial H/R injury. A series of in vitro experiments revealed the underlying mechanism of the AS/IV/miR-101a/*TGFBR1/TLR2*/MAPK pathway axis, which further explained why AS/IV is effective in myocardial H/R injury.

## Materials and methods

### Microarray analysis

The gene expression profile of E-MTAB-3573 was obtained from ArrayExpress (https://www.ebi.ac.uk/arrayexpress/experiments/E-MTAB-3573/). 23 AMI patients and 23 non-AMI healthy controls were selected for microarray analysis. According to results of the microarray analysis, 282 mRNAs were differentially expressed, and of these mRNAs, 143 mRNAs were upregulated, 139 were downregulated. The Limma package in R software was applied to screen the differentially expressed genes (DEGs). Data quality detection was conducted using a box plot, and quantile normalization was used for parallel experimental error elimination. An empirical Bayes method was used to search for significant DEGs,* P* < 0.05 and | log2 (Fold Change) |> 1.

### KEGG pathway enrichment analyses

To further analyze DEGs from microarray or genes from a Bioinformatics Analysis Tool for Molecular mechANism of Traditional Chinese Medicine (BATMAN-TCM), enrichment of the pathways was analyzed with GSEA v3.0 software. Pathway enrichment analysis was performed using the Kyoto Encyclopedia of Genes and Genomes (KEGG) pathway gene set. Default-weighted enrichment statistics ere adapted to conduct the permutation 1,000 times with normalized *P* < 0.05 considered to be significantly enriched.

### Cell culture and treatment

The human cardiomyocyte AC16 cell line was obtained from Dong Fang Hospital, Beijing University of Chinese Medicine (Beijing, China), then incubated with 90% DMEM-H (Dulbecco's modified eagle medium–High Glucose) with 10% FBS (Sigma, MO, USA). All of the cells were cultured in a cell incubator at a temperature of 37 °C, 95% air and 5% CO_2_. For stimulating the H/R injury, AC16 cells were cultured for 5 h with 5% CO_2_ and 1% O_2_ and 94% N_2_. Then, the hypoxia cells were moved to a normal culture environment to accomplish reoxygenation [28]. AS/IV concentrations of 20, 40, and 80 μM were used to treat cells.

### Quantitative real-time polymerase chain reaction (qRT-PCR)

TRIzol (Invitrogen, USA) was used for isolating total RNA from cells according to the manufacturer’s instructions. The qPCR Kits was used for miRNA (#A28007, Applied Biosystems), mRNA (#11,754,250, Invitrogen) amplification and total RNA detection (#F416XL, Invitrogen). The 2^−*∆∆Ct*^ method was utilized to normalize the relative quantification of mRNA, and GAPDH was applied for normalization. The GeneAmp™ PCR System 9700 (Applied Biosystems) was used to perform all of the reactions, which were repeated at least 3 times with the primers shown in Supplementary Table 1.

### Cell transfection

The pcDNA 3.1( +) vector (V79020) and miRNA mimic (MC12343) were purchased from Invitrogen (Invitrogen, Carlsbad, CA, USA). The sequences of the siRNAs were designed by https://rnaidesigner.thermofisher.com/rnaiexpress/, then synthesized by Sangon Biotech (Shanghai, China. Lipofectamine 2000 (Invitrogen, Carlsbad, CA) was used for cell transfection transfections, and the experiment was conducted according to the manufacturer's protocols. The sequences of the siRNAs are listed in Supplementary Table 1.

### Cell proliferation assay

A Cell Counting Kit-8 assay (CCK-8, Dojindo, Japan) was conducted to investigate cell proliferation. Cells were placed into 96-well plates with a 100 μL suspension in each well after inoculation, then cells were cultivated for 24 h in DMEM medium in a 5% CO_2_ incubator. The OD (optical density) values at 96 h were measured at 490 nm with the Varioskan Flash microplate reader (Thermo Fisher) after CCK-8 solution was added.

### LDH assay

2 × 104 AC16 cells were inoculated in 96-well plates and the cells were cultured for 12 h in a hypoxic environment. The medium was collected and centrifuged to obtain the supernatant. The LDH activity was measured by an LDH assay kit (Roche Diagnostics).

### MDA and SOD detection

The activity of superoxide dismutase (SOD) was detected by a kit (Jianglai, Nanjing, China). Similarly the malondialdehyde (MDA) activity was detected with a kit (Jianglai, Nanjing, China). The SOD activity was measured with the xanthine/xanthine oxidase (XOD) method. The MDA activity assay was measured by the thiobarbituric acid method. All experimental procedures are strictly followed the manufacturer's protocols.

### Quantification of cell apoptosis

The cells were stained with an apoptosis kit (Invitrogen, Carlsbad, CA, USA) was used to detect the percentage of AC16 cells in apoptosis according to the manufacturer’s instructions. The WinMDI software was used to distinguish and calculate the proportion of determine the number of viable cells, dead cells, early-stage and late-stage apoptotic cells after Annexin V/PI double staining.early-stage apoptotic cells, late-stage apoptotic cells and dead cells after double-stained with Annexin V/PI.

### Western blotting

Western blot was used to detect the expression levels of proteins. Total protein concentrations were determined using the bicinchoninic acid method. The BCA kit was purchased from Thermo Fisher. 30 microg of protein were separated by sodium dodecyl sulfate–polyacrylamide gel electrophoresis (SDS-PAGE) and the polyvinylidene difluoride (PVDF) membranes (Millipore, Billerica, MA) were used to transfer the proteins. For immunodetection, the membranes were cultivated overnight at 4 °C with the following primary antibodies: TGFBR1 (1:1000, ab31013, abcam), TLR2 (1:1000, ab213676, abcam), phosphor-ERK (1:1000, AP0472, ABclonal), ERK (1:1000, ab17942, abcam), phospho-p38 (1:1000, ab4822, abcam), p38 (1:1000, ab170099, abcam), Bax (1:1000, A0207, ABclonal), Bcl-2 (1:1000, A2845, ABclonal), Caspase-3 (1:1000, A2156, ABclonal), Cleaved Caspase-3 (1:1000, ab2302, abcam) and GAPDH (1:2500, ab9485, abcam). Next, the membranes were transferred to the second antibody goat anti-rabbit IgG H&L (HRP) (1:10,000, ab6721) and incubated for another 2 h on a shaking table. The Tanon 5200 chemiluminescence imaging system (Tanon, Shanghai, China) was used to scan the membranes. GADPH served as an internal control.

### Luciferase reporter assay

The relationships between *TGFBR1* or *TLR2* and miR-101a was predicted using StarBase (https://starbase.sysu.edu.cn/). The fragments from *TGFBR1* or *TLR2* were amplified through PCR, cloned into the firefly luciferase expression vectors pMIR-REPORT (Promega). The sequences of the hypothetical miRNA binding site were substituted as described. Lipofectamine 2000 (Promega) was used for the transient transfection of AC16 cells with the reporter vectors as well as the Renilla luciferase-expressing vector pRL-TK (Promega, Madison, WI, USA) and miR-101a mimic or NC. The cells were cultured for 48 h after transfection. Then, the relative luciferase reporter activity was detected.

### Statistical analysis

GraphPad Prism 6.0 was used for data analysis. The abovementioned experiments were conducted at least three times. Continuous data were documented as the mean ± standard deviation (SD). The differences between the two groups were analyzed with the Student’s *t*-test. A *P* value < 0.05 was regarded statistically significant.

## Results

### Identification of differentially expressed genes

According to results of the microarray analysis, 282 mRNAs were differentially expressed, and of these mRNAs, 143 mRNAs were upregulated, 139 were downregulated. A heatmap and a volcano plot were generated using the differentially dysregulated genes (Figs. S1, S2A). Then, we conducted a KEGG pathway (Kyoto Encyclopedia of Genes and Genomes) analysis of DEGs and genes obtained from BATMAN-TCM, and the results of the Venn diagrams show 6 common enriched signaling pathways (Figure S2B). The MAPK signaling pathway was selected as the object for subsequent experiments. Further analysis found CACNA1C, TLR2, FGF23 and TGFBR1 in the MAPK signaling pathway were included in both DEGs and BATMAN-TCM (Fig. S2C). Additionally, we used PubMed to screen TGFBR1 and TLR2 target miRNAs and 4 miRNAs (miR-92a, miR-19a, miR-101a and miR-29a) targeting both TGFBR1 and TLR2 were selected (Fig. S2D). Next, we measured the expression of 4 miRNAs in the H/R model of AC16 cells. Unlike the other 3 miRNAs, miR-101a was significantly downregulated in the H/R model of AC16 cells, so we chose it as the object of follow-up study (Fig. S2E). Then, the expression level of TGFBR1, TLR2 and miR-101a in the H/R cells was also verified by qRT-PCR (Fig. S2F).

### The effect of AS/IV on H/R in vitro

**Fig. 1:**
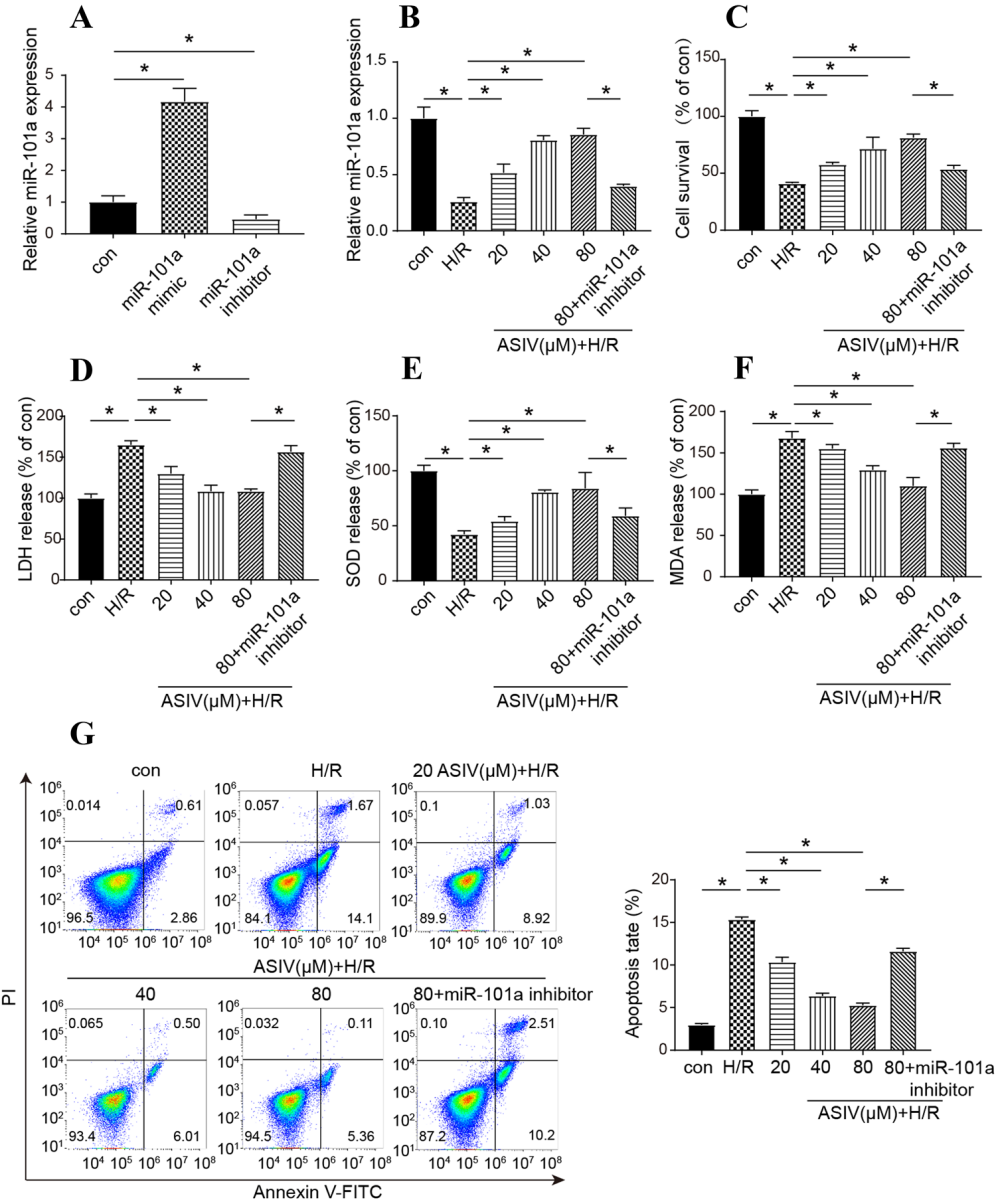
Effects of AS/IV on H/R in vitro cell experiments. There were 6 groups in these experiments including the con, 0 μM AS/IV+H/R cell, 20 μM AS/IV+H/R cell, 40 μM AS/IV+H/R cell, 80 μM AS/IV+H/R cell and 80 μM AS/IV+H/R+miR-101a inhibitor cell groups. **a** Efficiency of miR-101a mimic and inhibitor was verified by qRT-PCR. **b** Relative miR-101a expression was detected by qRT-PCR in these different groups. **c** Cell survival was detected by CCK-8 in these different groups. **d** LDH release was detected with an LDH assay kit in these different groups. **e** SOD activity was detected by DTPA in these different groups. **f** MDA activity was detected by a microplate spectrophotometer in these different groups. **g** Flow cytometry detected the apoptosis rate in these different groups. con: Control, H/R: hypoxia/reoxygenation. Data are shown as mean ± SD, *P < 0.05

To determine the effect of AS/IV, we performed a series of experiments in vitro. The experiments were divided into six groups: con, 0 μM AS/IV+H/R cell, 20 μM AS/IV+H/R cell, 40 μM AS/IV+H/R cell, 80 μM AS/IV+H/R cell and 80 μM AS/IV+H/R+miR-101a inhibitor cell group. First of all, the efficiency of miR-101a mimic and inhibitor was verified by qRT-PCR (Fig. 1a). We constructed the H/R model of AC16 cells and treated cardiomyocytes with different concentrations of AS/IV, and the results of qRT-PCR showed AS/IV could improve the expression of miR-101a in H/R cells (Fig. 1b). The cell survival (Fig. 1c) and SOD release (Fig. 1e) increased as the AS/IV concentration increased, whereas LDH release (Fig. 1d), MDA release (Fig. 1f) and apoptosis rates (Fig. 1g) were reduced compared to the con group. More importantly, the addition of miR-101a inhibitor counteracts the protective effect of AS/IV on cell viability and apoptosis. At the same time, the LDH, SOD and MDA levels were reversed partially. These results indicated that AS/IV had a significant protective effect on cardiomyocytes after H/R, and the contribution of miR-101a increase after AS/IV treatment which suggested that AS/IV might act by affecting the expression of miR-101a.

### MiR-101a targeted *TGFBR1* to influence H/R

**Fig. 2:**
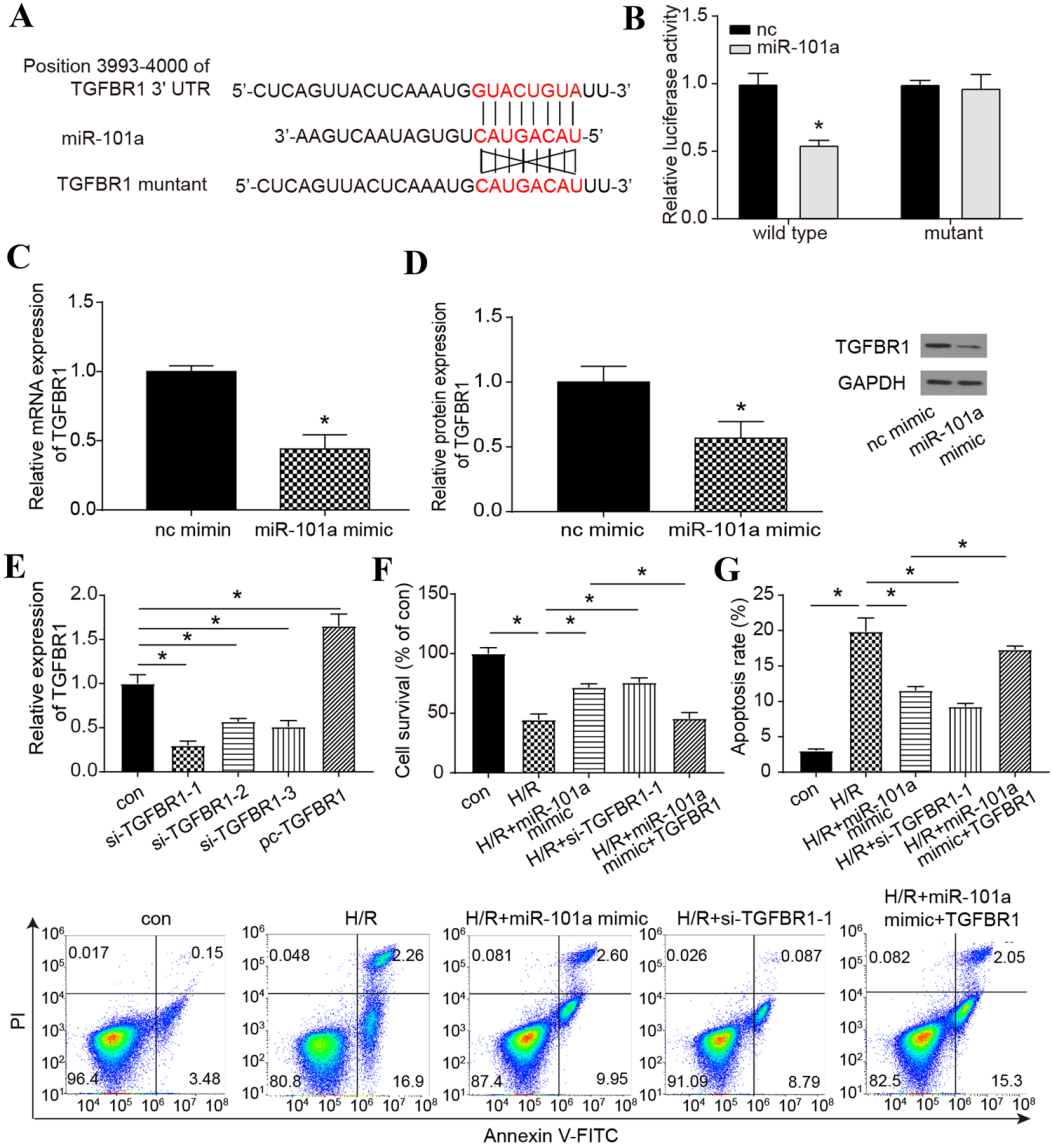
Regulating relationship between miR-101a and *TGFBR1* in H/R cells. **a**, **b** Targeting relationship of miR-101a and *TGFBR1*-wt/mut groups was detected by luciferase reporter assay*.***c** Relative expression of *TGFBR1* regulated by miR-101a mimic was detected by qRT-PCR. **d** Protein level of TGFBR1 regulated by miR-101a mimic was detected by Western blot. **e** Relative expression of *TGFBR1* in con, si-*TGFBR1-1,* si-*TGFBR1-2,* si-*TGFBR1-3* and pc-*TGFBR1* groups was detected with qRT-PCR. (F) CCK-8 detected cell survival in the con, H/R, H/R+miR-101a mimic, H/R+si-*TGFBR1,* H/R+miR-101a mimic+*TGFBR1* groups. (G) The apoptosis rate was detected by flow cytometry in the con, H/R, H/R+miR-101a mimic, H/R+si-*TGFBR1,* H/R+miR-101a mimic+*TGFBR1* groups. nc: normal control, con: Control, H/R: hypoxia/reoxygenation. Data are shown as mean ± SD, *P < 0.05

To verify the targeting relationship between miR-101a and *TGFBR1* in H/R cells, a dual-luciferase reporter assay was applied. The result indicated that miR-101a mimic could decrease the relative luciferase activity in *TGFBR1*-wt but had no effects on *TGFBR1*-mut (Fig. 2a, b), which implied that miR-101a could target *TGFBR1* in H/R cells*.* The results of qRT-PCR and Western blot analysis also showed that miR-101a mimic could significantly inhibit the expression of mRNA and protein level of *TGFBR1* (Fig. 2c, d). Then, we designed siRNAs (*si-TGFBR1-1, si-TGFBR1-2, si-TGFBR1-3*) and pc*-TGFBR1* to regulate *TGFBR1* expression. The relative expression levels of *TGFBR1* were increased by pc-*TGFBR1* and decreased by siRNAs (Fig. 2e). As si-*TGFBR-1* had the greatest inhibitory effect on *TGFBR1*, it was selected to complete the following experiments. Both of these results suggested that siRNAs, pc-RNAs and miRNA mimic were well-established (Fig. 2e). Additionally, miR-101a mimic and si-*TGFBR-1* significantly increased the proliferation of H/R cells. In addition, pc-*TGFBR* can counteract the effects of miR-101a mimic (Fig. 2f). However, the apoptosis rate displayed the opposite trend (Fig. 2g). These experiments have proven that miR-101a could affect the survival of H/R cells by affecting *TGFBR1*.

### MiR-101a targeted *TLR2* to influence H/R

**Fig. 3:**
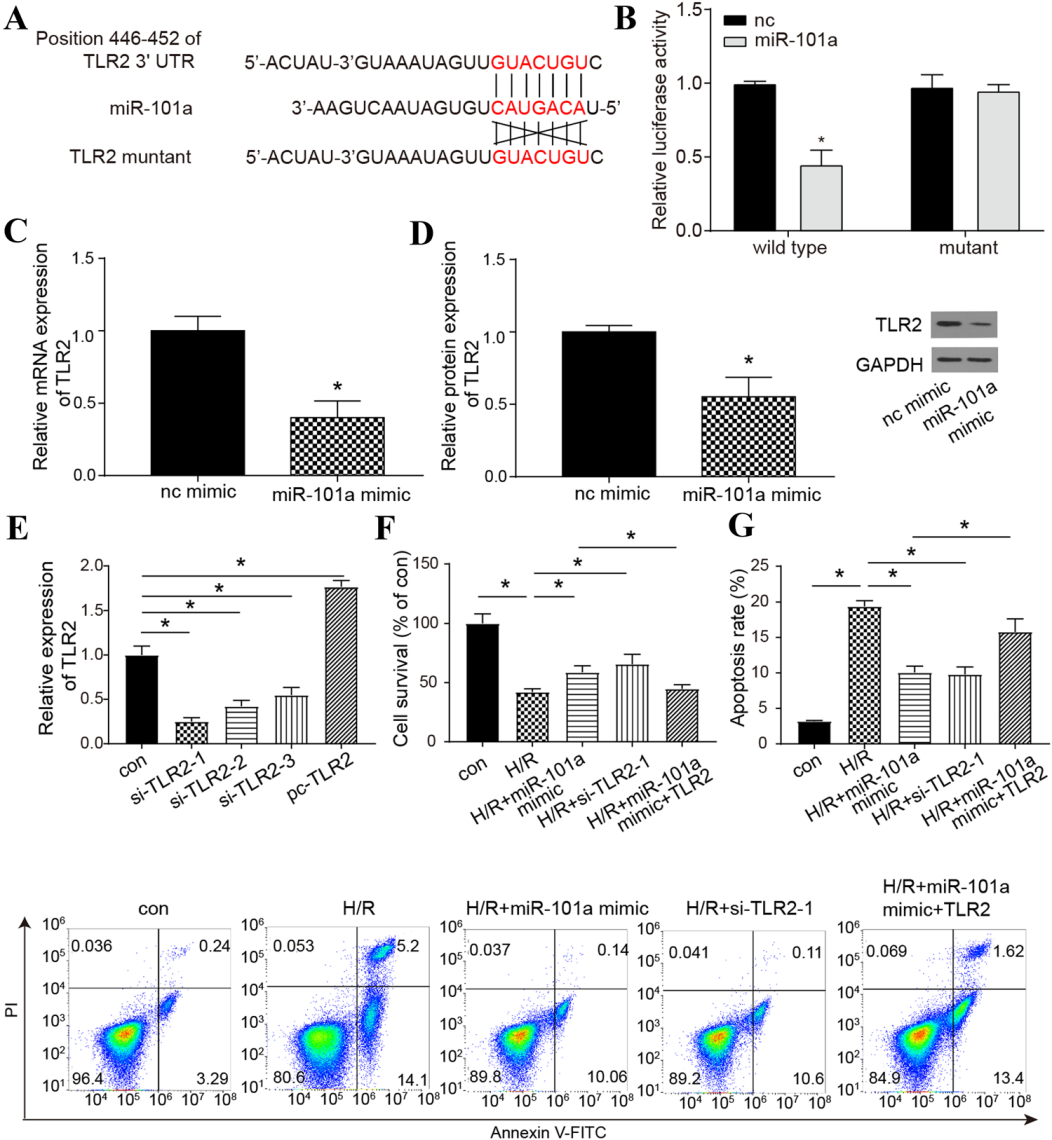
Regulating relationship between miR-101a and *TLR2* in H/R cells. **a**, **b** Targeting relationship of miR-101a and *TLR2*-wt/mut groups was detected by a dual-luciferase reporter assay*.***c** Relative expression of *TLR2* regulated by miR-101a mimic was detected by qRT-PCR. **d** Protein level of TLR2 regulated by miR-101a mimic was detected by Western blot. **e** Relative expression of *TLR2* in con, si-*TLR2-1,* si-*TLR2-2,* si-*TLR2-3* and pc-*TLR2* groups was detected with qRT-PCR. **f** CCK-8 detected cell survival in the con, H/R, H/R+miR-101a mimic, H/R+si-*TLR2-1,* H/R+miR-101a mimic+*TLR2* groups. **g** Apoptosis rate was detected by flow cytometry in the con, H/R, H/R+miR-101a mimic, H/R+si-*TLR2-1,* H/R+miR-101a mimic+*TLR2* groups. nc: normal control, con: Control, H/R: hypoxia/reoxygenation. Data are shown as mean ± SD, *P < 0.05

A dual-luciferase reporter assay was applied to determine the targeting relationship between *TLR2* and miR-101a in H/R cells. The results suggested that miR-101a mimic could decrease the relative luciferase activity in *TLR2*-wt, but it had no effects on *TLR2*-mut (Fig. 3a, b), which implied that miR-101a could target *TLR2.* Western blot and qRT-PCR results showed miR-101a mimic could significantly inhibit the mRNA and protein level of *TLR2* (Fig. 3c, d). SiRNAs (*si-TLR2-1, si-TLR2-2,* and *si-TLR2-3*) and pc*-TLR2* were applied to regulate *TLR2* expression, and the relative expression of *TLR2* was upregulated by pc-*TLR2* and downregulated by siRNAs. As si-*TLR2-1* had the best inhibitory effect on *TLR2*, it was selected to complete the following experiments (Fig. 3e). Furthermore, miR-101a mimic and si-*TLR2-1* significantly increased the proliferation of H/R cells. In addition, pc-*TLR2* can counteract the effects of miR-101a mimic (Fig. 3f). However, the apoptosis rate displayed the opposite trend (Fig. 3g). The above experiments have proved that miR-101a could affect the life process of H/R cells by affecting *TLR2*.

### AS/IV protects cell viability and apoptosis through the targeting effect of miR-101a on *TGFBR1* and *TLR2*

**Fig. 4:**
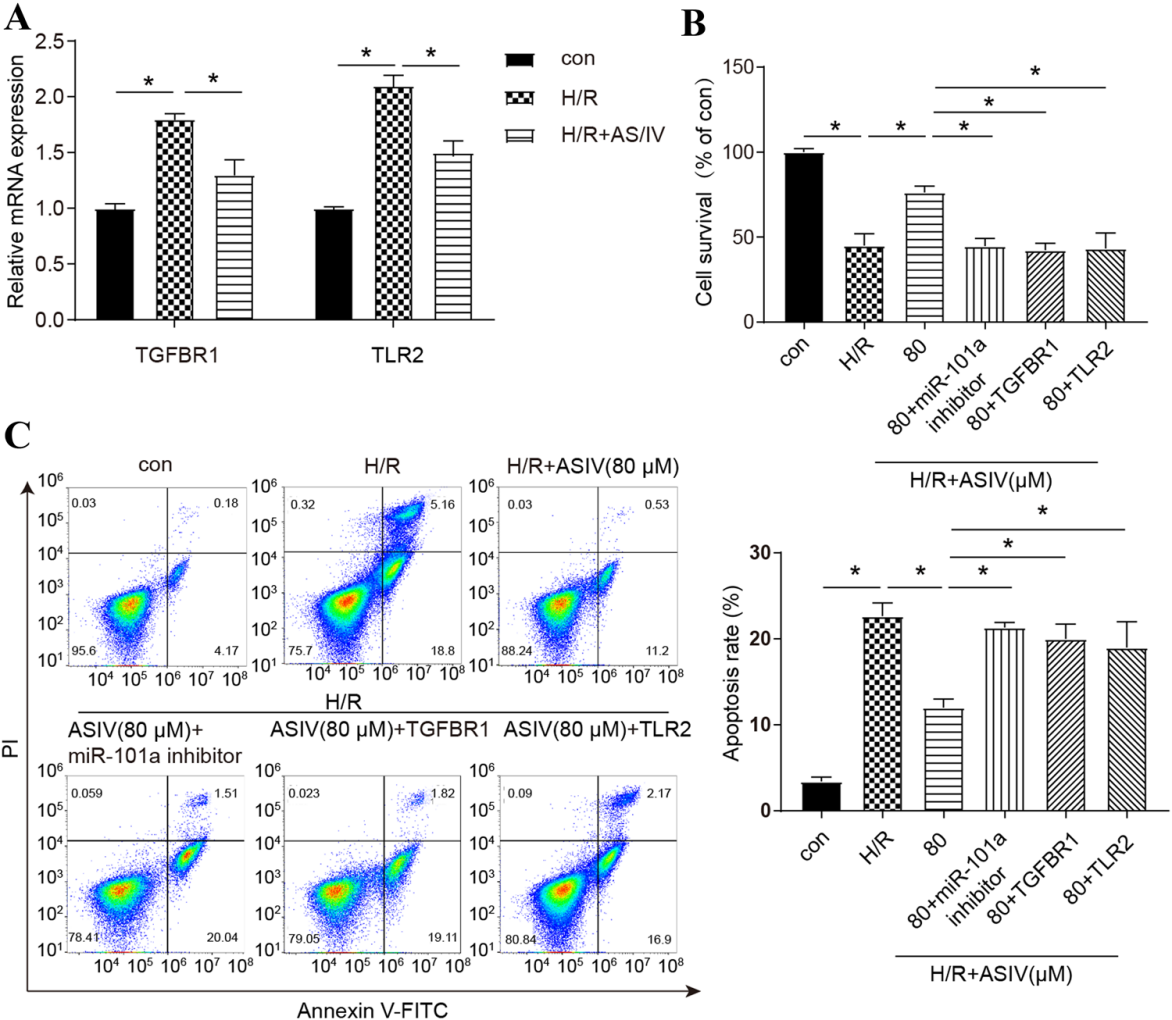
AS/IV protects cell viability and apoptosis through the targeting effect of miR-101a on *TGFBR1* and *TLR2*. **a** Relative expression of TGFBR1 and *TLR2* regulated by AS/IV was detected by qRT-PCR. **b** CCK-8 detected cell survival in the con, H/R, H/R+AS/IV (80 μm), H/R+AS/IV (80 μm)+miR-101a inhibitor*,* H/R+AS/IV (80 μm)+*TGFBR1*, H/R+AS/IV (80 μm)+*TLR2* groups. **c** Apoptosis rate was detected by flow cytometry in the con, H/R, H/R+AS/IV (80 μm), H/R+AS/IV (80 μm)+miR-101a inhibitor*,* H/R+AS/IV (80 μm)+*TGFBR1*, H/R+AS/IV (80 μm)+*TLR2* groups. con: Control, H/R: hypoxia/reoxygenation. Data are shown as mean ± SD, *P < 0.05

After determining the target regulatory relationship of *TLR2* and *TGFBR*1 by miR-101a, we intend to further identify the contribution of TGFBR1 and TLR2 in AS/IV-mediated beneficial effect in H/R injury. First of all, we found that AS/IV could downregulate the relative expression of *TGFBR1* and *TLR2* in the H/R cells by qRT-PCR (Fig. 4a). Then we found out that the miR-101a inhibitor reversed the positive effect of AS/IV on cell viability, moreover, the overexpression of *TGFBR1* and *TLR4* with AS/IV treatment which also negates the effect of AS/IV alone (Fig. 4b). Additionally, the flow cytometry also demonstrated that both miR-101a, *TGFBR1*, and *TLR2* could reverse the anti-apoptotic effect of AS/IV on cardiomyocytes (Fig. 4c). We believe that the above experimental results can prove that AS/IV plays a protective role against H/R injury by upregulating the expression level of miR-101a, which could further inhibit *TGFBR1* and *TLR2* through the targeting effect in vitro.

### Blocking the MAPK signaling pathway in H/R cells

**Fig. 5:**
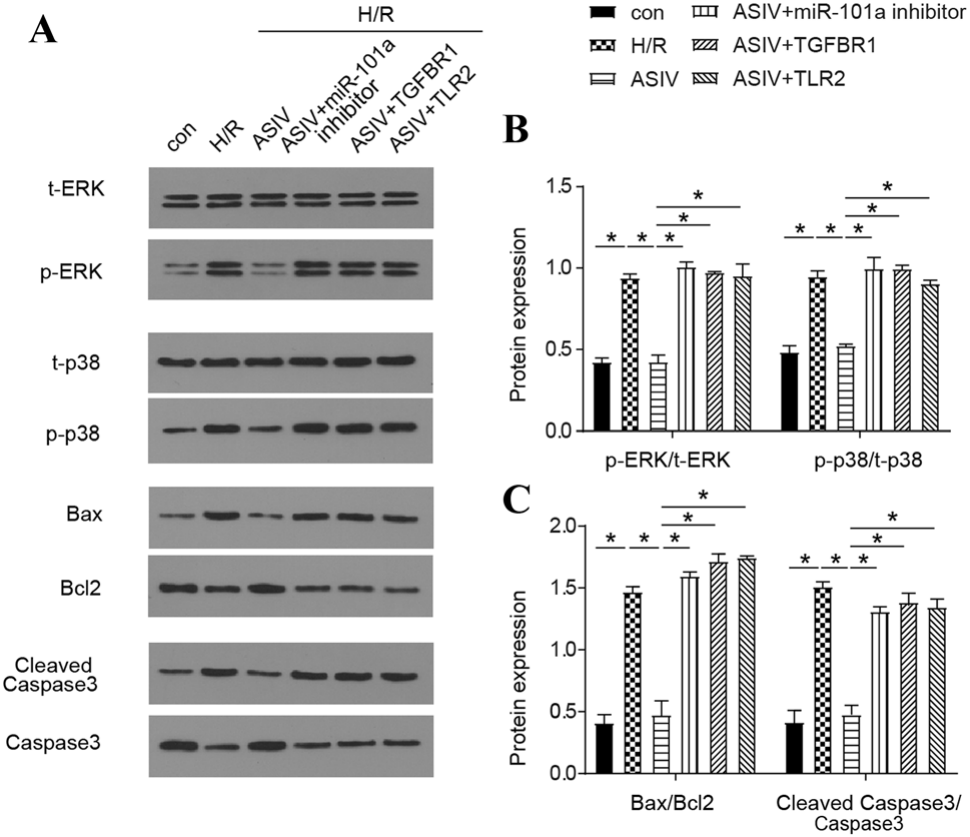
AS/IV reduces the expression of apoptosis-related proteins by regulating the MAPKs signaling pathway. **a**–**c** Expression level of MAPK signaling pathway proteins and apoptosis-related proteins (t-ERK, p-ERK, t-P38, p-P38, Bax, Bcl-2, Cleaved Caspase-3 and Caspase-3) was detected by Western blot in the con, H/R, H/R+AS/IV (80 μm), H/R+AS/IV (80 μm)+miR-101a inhibitor*,* H/R+AS/IV (80 μm)+*TGFBR1*, H/R+AS/IV (80 μm)+*TLR2* groups. con: Control, H/R: hypoxia/reoxygenation. Data are shown as mean ± SD, *P < 0.05

To investigate changes in the MAPK pathway, Western blot analysis was performed to detect the protein expression of ERK and p38 in the MAPK pathway and the expression of apoptosis-related proteins Bax, Bcl-2 and Caspase-3. The results indicated that the expression of p-ERK, p-p38, and Bax/Bcl-2 ratio and cleaved caspase-3/caspase-3 ratio were remarkably increased in H/R cells compared to the con group; meanwhile, the levels of these proteins gradually returned to normal by the treatment with AS/IV. However, the introduction of miR-101a inhibitor, *TGFBR1* and *TLR2* all reversed the effect of AS/IV (Fig. 5a–c). The above data indicate that AV/SI can play a positive role by influencing MAPK signaling pathway.

## Discussion

We observed and demonstrated the positive efficacy of AS/IV in the treatment of myocardial H/R injury in the current study. Furthermore, we finally uncovered the potential mechanism of AS/IV with miR-101a/*TGFBR1*/*TLR2* axis in the MAPK signaling pathway. AS/IV upregulated miR-101a expression and downregulated *TGFBR1* and *TLR2*. In addition, we found the MAPK pathway was significantly suppressed by AS/IV.

Through microarray analysis and retrieving concerned literatures, we determined that *TLR2* and *TGFBR1* were the mRNAs we were interested in, and the results of the analysis indicated that they were closely related to AS/IV and H/R injury. Then, we used PubMed to screen TGFBR1 and TLR2 target miRNAs, and we found that miR-101a was one of the miRNAs that had a targeting relationship with both *TLR2* and *TGFBR1* (Supplementary Fig. 2D)*.* Moreover, as shown in Supplementary Fig. 2F, miR-101a was abnormally expressed in the hypoxia/reoxygenation cell model. In the subsequent experiments, we demonstrated the targeting relationship between miR-101a and TLR2 and TGFBR1 through luciferase reporting experiments (Figs. 2b, 3b), which was in consistent with the previous studies [23, 29].

According to prior studies, AS/IV has already shown a positive effect on improving myocardial H/R via different methods. For instance, a study by Si e*t al.* suggested that postischemia treatment with AS/IV could attenuate H/R by upregulating HIF-1a expression. In addition, AS/IV could reduce the number of apoptotic cardiomyocytes with H/R [30]. A meta-analysis showed AS/IV significantly reduced myocardial infarction area and protected heart function in mice compared to the control group [31]. Another similar report also indicated that AS/IV could improve myocardial H/R in rats by suppressing calcium-sensing receptor-mediated apoptotic signaling pathways [32]. Consistent with research mentioned above, our results showed that AS/IV could strengthen cell viability, reduce apoptosis, and the promotional effect was more significant as the concentration of AS/IV solution increased. In addition, both promotion of SOD activity and the decline of MDA and LDH emphasized that AS/IV could attenuate myocardial H/R injury. Furthermore, AS/IV treatment significantly increased the expression level of miR-101a.

MiR-101a was reported downregulated in infarcted heart tissue [33], and the level of miR-101a was significantly reduced following H/R treatment of H9c2 cardiomyocytes [34]. In our present study, we detected that miR-101a was decreased in the H/R AC16 cells, which was in consistent the previous study [35, 36]. However, AS/IV treatment with concentration gradient can upregulate the expression of miR-101a. Therefore, our results confirmed that AS/IV could induce miR-101a overexpression in myocardial H/R, and increase the cell viability and suppress cell apoptosis.

The negative relationship between *TGFBR1* and miR-101a has been reported in mouse livers hepatic stellate cells [29]. Here, we conducted a dual-luciferase assay and qRT-PCR to verify the relationship that miR-101a decreased TGFBR1 expression in myocardial H/R. To demonstrate that miR-101a plays a protective role in cardiomyocytes by negatively regulating TGFBR1, a recovery experiments were carried out. Our experimental results are consistent with our hypothesis, the protective effect of miR-101a on H/R-induced apoptosis was reversed by *TGFBR1* overexpression. Similar to our results, Zhang et al*.* found that glutamine protected cardiomyocytes from hypoxia/reoxygenation injury through inhibiting TGFBR1/2-Smad3 pathway under high-glucose (H/G) conditions. The expression level of TGFβ significantly increased in the H/G+H/R group [37]. Meanwhile, the activation of the TGFβ signaling pathway can also promote cardiomyocyte hypertrophy and heart failure [15]. At the same time, the microRNA-15 family has been reported to regulate myocardial hypertrophy and fibrosis by targeting TGFBR1 [38]. Taken together, we believe that TGFBR1 has a negative effect on the treatment of myocardial H/R injury, and this effect is regulated by miR-101a to some extent.

Furthermore, we concluded the targeting relationship between miR-101a and *TLR2* through StarBase analysis and a dual-luciferase reporter assay. In addition, in cell apoptosis detection, we discovered that the overexpression of *TLR2* counteracted the anti-apoptosis effect of miR-101a on AC16 cell apoptosis. A growing number of papers have reported that *TLR2* plays a key regulatory role in heart disease. For instance, Selejan et al. found that the expression of TLR2 positive monocytes and inflammatory cytokines in the blood of patients with myocardial infarction was upregulated [39]. The finding means that monocytes are recruited into the heart to play a negative role. However, Favre et al. reported that in a mouse model of myocardial IR injury, *TLR2* knockdown significantly reduced the area of myocardial infarction [40]. Judging from the results of our experiment, we have observed that AS/IV treatment significantly attenuated H/R-induced apoptosis of cardiomyocytes and increased cell viability, while the expression of miR-101a was increased. Moreover, the expression of both *TGFBR1* and *TLR2* was decreased. More importantly, we found that the protective effect of AS/IV was offset by overexpression of TGFBR1 and TLR2 in AS/IV-treated hypoxia/reoxygenation cells. Therefore, the targeting of *TGFBR1* and *TLR2* by miR-101a may be a mechanism for increase of cell viability and attenuation of myocardial apoptosis during myocardial H/R injury by treatment of AS/IV.

In the last stage of the experiment, we detected the activation of MAPK signaling pathway and the expression levels of apoptosis-related proteins by Western blot. As shown in Fig. 5, the results indicated that the expression of p-ERK and p-p38 were remarkably increased in H/R cells, suggesting that the MAPK signaling pathway was activated. However, the addition of AS/IV inhibited the MAPK signaling pathway (Fig. 5b). Bax/Bcl-2 ratio and cleaved caspase-3/caspase-3 ratio were remarkably increased in H/R cells; meanwhile, the levels of these proteins gradually returned to normal by the treatment with AS/IV. Most importantly, the overexpression of TGFBR1 and TLR2 also reversed the effects of AS/IV and activated the MAPK signaling pathway. These experimental data indicate that AV/SI can play a protective role by affecting MAPK signaling pathway.

There were some limitations that should be taken into consideration. Firstly, we only conducted the experiments in vitro, while animal experiments were omitted. Additionally, we only selected human cardiomyocyte AC16 cells for the present research. The neonatal rat or mouse cardiomyocytes and human cardiomyocytes were not included in our design. Therefore, we will continue to explore the specific molecular mechanism of primary cardiomyocytes in the future.

## Conclusion

Our study shows that AS/IV had the capacity to attenuate cardiomyocytes H/R injury via the miR-101a/*TGFBR1*/*TLR2* axis through the MAPK signaling pathway. As for possible future research directions, we seek to determine whether AS/IV can protect myocardial H/R injury through additional mechanisms. Despite its hypotoxicity for cardiomyocytes at a dilute concentration, AS/IV might act as an effective modulator and protect cardiomyocytes against myocardial H/R injury. Hence, the results of this study also offer meaningful information for developing new drugs that target miR-101a/*TGFBR1*/*TLR2* to treat myocardial H/R injury.

## Electronic supplementary material

Below is the link to the electronic supplementary material.

Supplementary file1 (DOCX 15 kb)

Supplementary file2 (TIF 12332 kb)

Supplementary file3 (TIF 11543 kb)
