# Supplementary material for: Astragaloside IV protects human cardiomyocytes from hypoxia/reoxygenation injury by regulating miR-101a
Source: Mol Cell Biochem. 2020 May 11;470(1):41–51. doi: 10.1007/s11010-020-03743-5 (PMC7272390; doi:10.1007/s11010-020-03743-5)
Supplement: Supplementary file 1 — Supplementary file1 (DOCX 15 kb) [file 11010_2020_3743_MOESM1_ESM.docx]

**Supplementary Table 1.** The sequences of primers and siRNAs

| Gene | Sequence |
| --- | --- |
| TGFBR1 | F:5’- TCGTCTGCATCTCACTCAT-3’ |
|  | R:5’- GATAAATCTCTGCCTCACG-3’ |
| TLR2 | F:5’-TTGCTCCTGTGACTTCCTGTC-3’ |
|  | R:5’-GAGCGTCACAGCGGTAGC-3’ |
| U6 | F:5′-ATTGGAA CGATACAGAGAAGATT-3′ |
|  | R:5′-GG AACGCTTCACGAATTTG-3′ |
| GAPDH | F:5’-TGCACCACCAACTGCTTA GC-3’ |
|  | R:5’-GGCATGGACTGTGGTCATGAG-3’ |
| si-TGFBR1-1 | 5’-CAUUCACCAUCGAGUGCCAAAUGAA-3’ |
| si-TGFBR1-2 | 5’-CCAACUACUGUAAAGUCAU-3’ |
| si-TGFBR1-3 | 5’-GCUUGUUCAGAGAACAAUU-3’ |
| si-TLR2-1 | 5’-UGAAGCAUCAAUCUCAAGUUCCUCA-3’ |
| si-TLR2-2 | 5’-GGUAAAGUGGAAACGUUAA-3’ |
| si-TLR2-3 | 5’-GGAAGAUAAUGAACACCAA-3’ |
