# Supplementary figures and images for: Astragaloside IV protects human cardiomyocytes from hypoxia/reoxygenation injury by regulating miR-101a
Source: Mol Cell Biochem. 2020 May 11;470(1):41–51. doi: 10.1007/s11010-020-03743-5 (PMC7272390; doi:10.1007/s11010-020-03743-5)

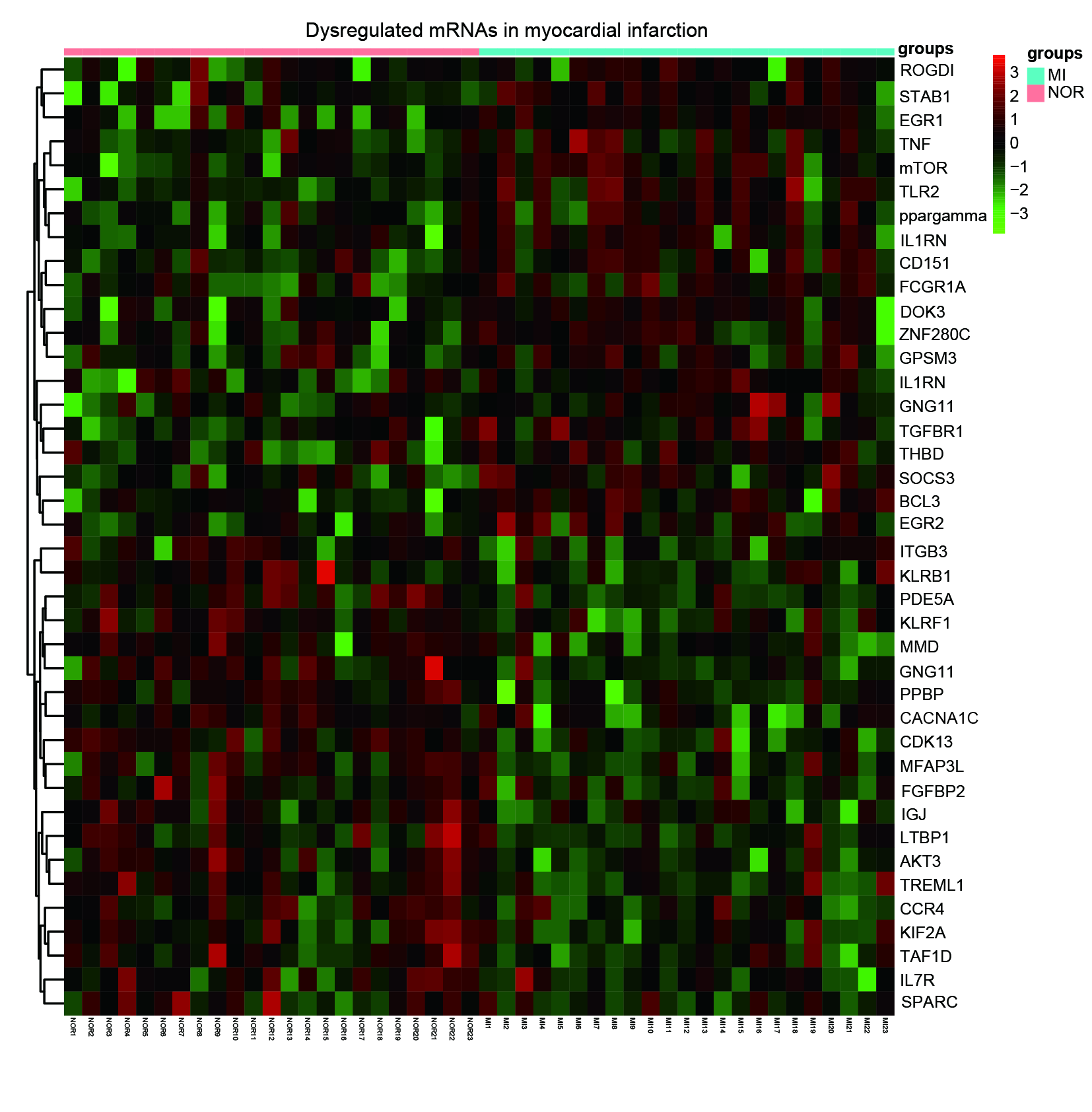

Supplement: Supplementary file 2 — Supplementary file2 (TIF 12332 kb) [file 11010_2020_3743_MOESM2_ESM.tif]

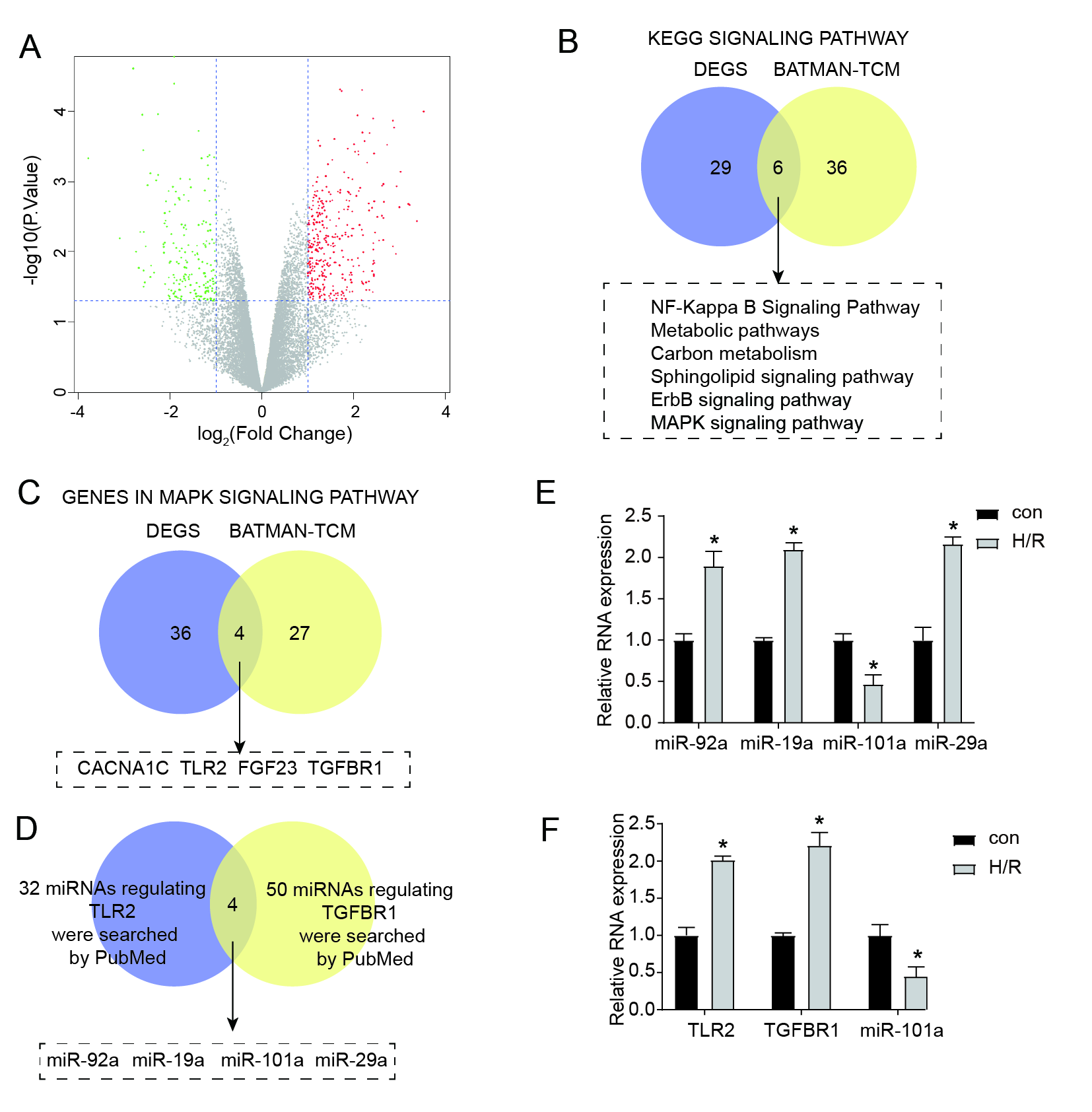

Supplement: Supplementary file 3 — Supplementary file3 (TIF 11543 kb) [file 11010_2020_3743_MOESM3_ESM.tif]
